# Socioeconomic Patterning of Childhood Overweight Status in Europe

**DOI:** 10.3390/ijerph9041472

**Published:** 2012-04-16

**Authors:** Cécile Knai, Tim Lobstein, Nicole Darmon, Harry Rutter, Martin McKee

**Affiliations:** 1 London School of Hygiene & Tropical Medicine, 15–17 Tavistock Place, London WC1H 9SH, UK; Email: harry.rutter@lshtm.ac.uk (H.R.); martin.mckee@lshtm.ac.uk (M.M.); 2 International Association for the Study of Obesity, Charles Darwin House, 12 Roger Street, London WCIN 2JU, UK; Email: tlobstein@iaso.org; 3 INRA, UMR1260, INSERM, UMR1062, Nutrition, Obesity and Risk of Thrombosis, Faculté de Médecine, Aix-Marseille University, F-13385, Marseille, France; Email: nicole.darmon@univ-amu.fr

**Keywords:** overweight, social gradient, child, Europe

## Abstract

There is growing evidence of social disparities in overweight among European children. This paper examines whether there is an association between socioeconomic inequality and prevalence of child overweight in European countries, and if socioeconomic disparities in child overweight are increasing. We analyse cross-country comparisons of household inequality and child overweight prevalence in Europe and review within-country variations over time of childhood overweight by social grouping, drawn from a review of the literature. Data from 22 European countries suggest that greater inequality in household income is positively associated with both self-reported and measured child overweight prevalence. Moreover, seven studies from four countries reported on the influence of socioeconomic factors on the distribution of child overweight over time. Four out of seven reported widening social disparities in childhood overweight, a fifth found statistically significant disparities only in a small sub-group, one found non-statistically significant disparities, and a lack of social gradient was reported in the last study. Where there is evidence of a widening social gradient in child overweight, it is likely that the changes in lifestyles and dietary habits involved in the increase in the prevalence of overweight have had a less favourable impact in low socio-economic status groups than in the rest of the population. More profound structural changes, based on population-wide social and environmental interventions are needed to halt the increasing social gradient in child overweight in current and future generations.

## 1. Introduction

A social gradient in overweight runs through European and other developed countries, with those who are poorest the most likely to be overweight [1,2,3,4,5,6,7,8,9]. Although the prevalence of overweight has increased across the population of these countries, the rise has not been as severe among the wealthiest parts of the population, leading to widening social disparities among adults [4,8,10,11,12,13,14,15,16]. The reasons underpinning these trends are varied, and differ across contexts and cultures. It is possible that greater structural inequalities within a society lead those in the most deprived groups to experience greater daily financial and other constraints; this in turn may adversely influence their opportunities for an active, healthy lifestyle [17]. Equally, obesity prevention interventions (and treatment) may be less successful among lower-income groups than amongst those with higher incomes [18]. Any obesity prevention interventions may well influence social categories differentially, and in those populations with the greatest disparities there may be larger numbers whom these interventions do not reach. There may also be a tendency for members of certain minority ethnic groups to have elevated levels of obesity. These trends may in part be due to socio-economic differences, including greater exposure to environments conducive to weight gain, but may also reflect culturally-specific health-related behaviour patterns [19,20,21] and, in a few cases, differences in genetic susceptibility [22].

The trends and patterns of social inequality in child overweight have, until recently, been less intensively explored even though the trajectory of childhood overweight prevalence in Europe has risen sharply in recent decades [23,24,25,26]. There have been some signs of levelling off [27,28,29,30,31] but also indications to the contrary, illustrated by a renewed rise in obesity in French children, after ten years of stabilization [32,33]. Despite these fluctuations and differences, the prevalence of child overweight (including obesity) in Europe is extremely high, particularly for children from socioeconomically deprived backgrounds [10,34,35,36]. Socioeconomic differences seem to emerge as early as three years of age [37]; Howe *et al.* [38] modelled the social patterning of obesity from 2 to 10 years and socioeconomic differentials in Body Mass Index (BMI) began to emerge at 4 years. 

**Figure 1:**
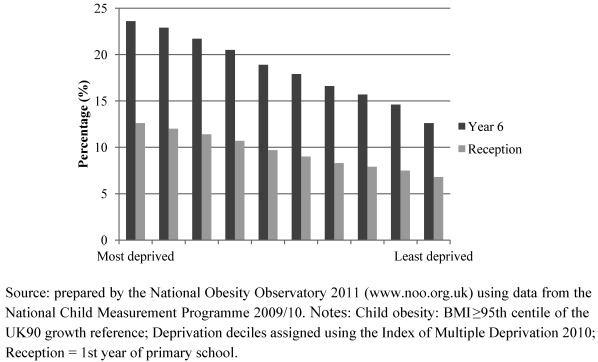
Prevalence of obesity by deprivation decile in English school children, in Reception and Year 6.

This may be a relatively recent phenomenon. In an analysis of the 1958 British Birth Cohort and their offspring, Pinot de Moira *et al. *[39] found negligible socioeconomic inequality in childhood obesity in 1958, but among the offspring of these individuals (born between 1982 and 1987), higher socioeconomic status was associated with lower adiposity. Moreover the social patterning of childhood obesity exists irrespective of whether child weight is self-reported or measured. The 2005/6 WHO Health Behaviour in School-aged Children Survey reported a significant negative association between family affluence and self-reported overweight and obesity, with children from more deprived families more likely to be obese or overweight, especially in Western Europe [35]. A 2008 systematic review of studies on the association between socioeconomic status and childhood overweight concluded that this pattern now predominates in wealthier European countries [34]. Many of these countries have been monitoring their data over recent years. Some of the most vivid examples come from England’s National Child Measurement Programme 2009/2010, which suggests that obesity prevalence among the most deprived 10% of Reception (year 1 of school) and Year 6 children is approximately twice as high as for among in the least deprived 10% (Figure 1). 

This paper asks two questions. First, is there an association between socioeconomic inequality and prevalence of child overweight in European countries? Second, is there evidence that social disparities in child overweight are changing over time? We address the first question by means of a cross-sectional comparison of data on inequality and overweight, and the second question by means of a review of published literature. We conclude by discussing some of the potential explanatory factors for the widening of the social gradient in child overweight.

## 2. Methods

### 2.1. Cross-Country Comparisons

There are many different ways of expressing inequalities. For this work information on the degree of inequality in national populations was obtained from the UNICEF report on the wellbeing of children in economically advanced countries in which the authors consider what they term the ‘relative inequality in income in households with children’ for the year 2008 [40]. Details of the methods used are given in the report, which provides data for 22 European countries. This is the gap between the median and the 10th percentile of household incomes for households with children, expressed as a percentage of the median income. This approach captures the magnitude of the lower tail of the distribution, without introducing distortions that may be caused by small number of extreme values [40].

National data for overweight in children as near to the year 2008 as possible were obtained from two sources. The first provides the combined overweight prevalence derived from children’s self-reported heights and weights in the Health Behaviour of School Children Study [35] which included 35 European surveys conducted over the period 2005–2006. Data for children aged 11 years at the time of survey were taken for those countries for which household inequalities data were also available. Prevalence for boys and for girls was averaged for the present analysis. 

Self-reported data can be affected by reporting bias, including bias affected by inequality. A second set of overweight prevalence data was obtained from surveys of measured heights and weights, as reported by the OECD [41]. Although these data are collected from surveys undertaken in countries in different years, using different methodologies and different age groups, they are the best available data prior to and including 2008 for measured overweight prevalence in children in Europe. Both sources have strengths and weaknesses, with the first offering international comparability, but at the cost of potential reporting bias, while the second offers unbiased values but at the cost of loss of comparability of sampling.

In both surveys of children’s heights and weights, overweight was defined as Body Mass Index greater than a threshold cut-off for children recommended by the International Obesity Task Force and published by Cole *et al. *[42]. Analysis of the relation between overweight prevalence and household inequality used linear correlation for unadjusted data (Excel CORREL function © Microsoft Office).

### 2.2. Review of Literature on the Social Gradient in Childhood Overweight in European Countries

A standardised search strategy was applied across five databases: Pubmed, MEDLINE/Ovid, CAB Abstracts, EMBASE, and Web of Knowledge. Search terms included ‘children’, ‘obesity’, ‘overweight’, as well as ‘inequality’ (‘disparity’, ‘gap’, ‘social gap’, ‘socioeconomic inequality’); ‘increase’ (and synonyms); social gradient; and the list of European countries. The reference list of included studies and other relevant publications were also scanned for any references to potentially eligible studies not captured in the database searches. Only studies published in English were considered. Studies were included if they reported trends in the social gradient in overweight or overweight in child populations of European countries. Quality criteria for eligibility of the studies were inclusion of at least two time points, specified measure(s) of social stratification, explicitly defined population being studied (by age, geographical settings), and specified method of weight assessment (either self-reported or measured). Upon completion of the electronic searches, results were merged and duplicates removed. Citations were screened against the eligibility criteria. We present a narrative summary of the included studies in terms of study characteristics’ and results. 

## 3. Results

### 3.1. Results for Cross-Country Comparisons

Table 1 shows the data obtained for household income inequalities and overweight prevalence. The measure of household inequality ranged from 39.4 (Norway) to 56.6 (Greece). Self-reported heights and weights gave a range of overweight prevalence values from 5% of children (Switzerland) to 23% (Portugal). Measured heights and weights gave a range of overweight prevalence values between 9% (Slovakia) and 33% (Italy and Spain). The correlation between the two measures of overweight prevalence was r = 0.70 indicating some unexplained variation, and therefore both measures were separately compared with the measure of household inequality. 

**Table 1:** Cross country measures of income inequality and child overweight prevalence (%) in 22 European countries, ordered by income inequality.

	Income inequality *: households with children, 2008 ^a^ (%)	Overweight prevalence (self-reported, 2005-2006, age 11) ^b^	Overweight prevalence (measured, year and age-range stated) ^c^
Greece	56.6	18	22 (2003) 13–17
Portugal	56.2	23	32 (2003) 7–9
Spain	56	19	33 (2000) 13–14
Italy	54.1	20	33 (2006) 8–9
Poland	51.2	13	14 (2001) 7–9
Belgium	50.6	9 *	22 (2005) 4–15
United Kingdom	50.1	15 **	32 (2004/8) 5–17 **
Slovakia	48.9	10	9 (1999) 11–17
Germany	48.1	11	20 (2002) 5–17
Ireland	47.4	16	25 (2007) 4–13
Luxembourg	46.4	12	-
Hungary	44.6	17	19 (2005) 7–18
Switzerland	44.3	5	15 (2007) 6–13
Czech Republic	43.7	19	21 (2005) 6–17
Finland	41.9	18	-
France	41.6	10	19 (2006) 11–17
Netherlands	41.5	6	16 (2003) 5–16
Sweden	41.2	8	22 (2001) 6–13
Iceland	40.2	12	23 (2003) 9
Austria	40	11	20 (2003) 8–12
Denmark	39.7	10	14 (1997) 5–16
Norway	39.4	8	16 (2005) 3–17

Source: ^a^ UNICEF 2010 [40], ^b^ WHO Health Behaviour of School-age Children 2005/6 [35], ^c^ OECD [41]. * ‘relative inequality in income in households with children’ for the year 2008, based on the gap between the median and the 10th percentile of household incomes for households with children, expressed as a percentage of the median income value. ** unweighted average of two regions.

**Figure 2:**
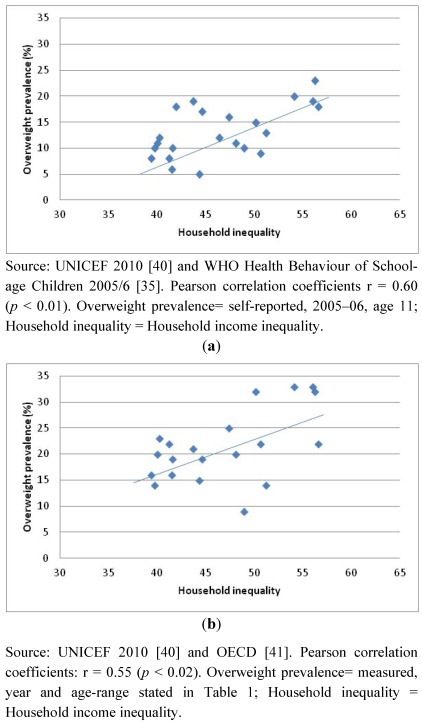
(**a**) Household income inequality by child self-reported overweight; (**b**) Household income inequality by child measured overweight.

Figure 2a and Figure 2b show the relation between household inequality and overweight prevalence using self-reported heights and weights (Figure 2a) and measured heights and weights (Figure 2b). These figures suggest that worsening household income inequality is positively associated with both self-reported and measured child overweight prevalence. Analyses using Pearson correlation coefficients gave values of r = 0.60 (*p* < 0.01) and r = 0.55 (*p* < 0.02) respectively. 

### 3.2. Variations in Childhood Overweight by Social Grouping over Time

#### 3.2.1. Description of Studies

The search strategy yielded 128 citations. After removing duplicates and screening, seven studies met the eligibility criteria [43,44,45,46,47,48,49], reporting trend data on child overweight prevalence and socio-economic status (SES) measures since 2000 and from European countries. The measures of child SES included occupation of the head of the household, parental education, social class, and composite scores. One study was conducted in each of Belgium [49] and Finland [44]; two in France [43,45]; and the remaining three in the UK [46,47,48]. 

#### 3.2.2. Influence of Socioeconomic Factors on the Distribution of Child Overweight over Time

Seven studies from four countries reported on the influence of socioeconomic factors in the distribution of child overweight over time (Table 2). Four [45,47,48,49] out of seven studies reported widening social disparities in childhood overweight, a fourth found statistically significant disparities only in a small subgroup [44], one found non-statistically significant disparities [46], and a lack of social gradient was reported in the seventh study [43]. 

Spiegelaere *et al*. [49] conducted a retrospective cohort study on a sample of young adolescents from the Brussels region, in five social groups defined according to parental profession, assessing their overweight status at age 12 and again at age 15; social inequalities increased between the two examinations, significantly so for the least advantaged social group. Romon *et al. *[45] compared two cross-sectional surveys of children in the final year of nursery school in the city of Lille (France), using parental occupation and social class as indicators of child socioeconomic status. Weight status of the 5-year-old study subjects differed as a function of social class over time: in 1989 there were no significant differences between social classes whereas in 1999 there was a clear difference between classes both for overweight. This was not confirmed by Lioret *et al. *[43] in their investigation of a social gradient in overweight among 3–14 year olds, using data from two cross sectional national food consumption surveys. Though the prevalence of childhood overweight was significantly and inversely correlated to all SES indicators, there was no change in social gradient of childhood overweight over time, from 1998 to 2007. Similarly, in Finland, Kautiainen *et al. *[44] investigated associations between time trends in adolescent overweight prevalence and socioeconomic status, analysing 14 successive Adolescent Health and Lifestyle Survey (AHLS) against a range of SES indicators. Overall there were no observed socioeconomic differences in overweight trends over time, though they did find a non-significant increasing trend in the prevalence of overweight in 12-year-old girls with the least educated mothers. In the UK, three studies indicate that the social gradient in childhood overweight may be increasing. In their analysis of 3 year-old entrants to the National Community Child Health Database over eleven years, Brunt *et al. *[46] found a non- statistically significant greater rate of increase of overweight in children from the most deprived areas compared with those from the least deprived. Semmler *et al. *investigated changes in child BMI over time with respect to parental weight status, comparing two sociodemographically similar subsets of the Twins Early Development Study [47]. They found an increasing social gradient in their cohort, with changes in adiposity from ages 4 to 11 in relation to familial SES: at age 11, significantly more children from more deprived families (29%) than less deprived families (17%) were overweight. Moreover their findings indicated a strong influence of parental weight. They found a significant interaction between parental weight status and familial socioeconomic status for change in BMI over time: among families with lean parents, changes in child overweight were similar across socioeconomic groups, however in families with obese parents children from lower SES families gained 0.70 BMI SD scores (SD scores or z-scores represents the deviation compared with an average child of the same sex and age) compared with a gain of only 0.24 BMI SD scores for children from higher SES families. In their analysis of annual Health Survey for England data from 1997 to 2007, Stamatakis and colleagues [48] reported an increasing and statistically significant social gradient in the overweight of 5–10 year old children over time. The socioeconomic position score gradient also increased over time, reaching statistical significance in 2006/7 for boys’ overweight, at which point the overweight prevalence in the most deprived group was twice than that in the least deprived groups. Similar gradients in girls’ overweight were found.

## 4. Discussion

### 4.1. Strengths and Limitations

This paper contributes to our understanding of social inequalities in child obesity in European countries, providing evidence of a widening social gap within child populations. 

A first limitation of our review is that we relied on published studies rather than analysing existing survey data from European countries directly. Second, while the data on self-reported heights and weights refer to a very narrow age range, those on measured heights and weights refer to children belonging to very different age groups, drawing on heterogeneous data sets gathered using different methods. This underscores the importance of collecting comparable data. A third limitation is that we do not know if the measured height and weight data were gathered on representative samples of children. The fourth limitation was the restriction of our focus to European countries. By failing to capture the global perspective on the social gradient in childhood obesity we may have missed out on identifying valuable contextual information.

### 4.2. Potential Explanations in Those Studies Showing a Widening of the Social Gradient in Childhood Obesity in Europe

Among the studies reviewed, four reported significant widening of the social gradient, while three found no significant change, or widening only in a sub-group. None found a significant narrowing. What factors might explain the widening gap where one is observed? Biological and behavioural risk factors for overweight exist at every stage of life and are exacerbated by socioeconomic deprivation [50]. These relationships and effects are complex, often intergenerational [51], and likely grounded in parental risk factors [52,53,54,55]. 

Parental overweight is identified as one of the strongest risk factors for childhood obesity [47,56,57]. Maternal weight also has an important influence on child outcomes [58]: an underweight pregnant woman influences the likelihood of obesity in later life for the fetus via mechanisms involved in *in utero* programming [59] and an overweight pregnant woman has greater risk of delivering a high birth-weight infant (>4,000 g) which in turn can increase the risk of childhood and adult obesity [59,60]. 

Behavioural risk factors, most notably maternal smoking during pregnancy, have also been shown to confer added long-term risk to children, combining low birth weight with later weight gain [53,61]. A social gradient in smoking has been observed during the third trimester of pregnancy, in France, with pregnant women in lowest level occupations smoking increasingly from 1995–2003 but women in management positions smoking less over the same period [62,63,64]. Mothers in more deprived circumstances are also less likely to breastfeed their newborns [53], and to introduce unmodified cow's milk too early [65,66]. This is likely to increase inequalities as breastfeeding confers protection against childhood obesity [67]. Moreover, women who are already overweight before pregnancy are less likely to initiate breastfeeding and more likely to discontinue breastfeeding earlier than do normal weight women [68,69,70,71]. 

**Table 2:** Characteristics of seven included studies.

ID	Country	Years range	Study design	Characteristics of study subjects	Indicators of child SES	Results
Brunt 2008 [46]	UK	1995–2005	National Community Child Health Database (NCCHD), over 11 years	N = 3756 – 4548 per year 3-year-old children in South Wales	Townsend Material Deprivation Score, which comprises assessments of unemployment, overcrowding, home and car ownership	Non- statistically significant greater rate of increase of overweight in children from the most deprived areas compared with those from the least deprived.
Kautiainen *et al. *2009 [44]	Finland	1979–2005	14 cross-sectional surveys, the Adolescent Health and Lifestyle Survey (AHLS) (biennial)	N = 3105 – 8390 per year 12–18 year olds (nationally representative sample)	Family structure (1 or 2 parents), degree of urbanization, area of residence, father or guardian’s occupational status, school achievement, mother and father’s employment status, mother’s education level	The increase in the prevalence of overweight over time did not differ between age groups. However an increasing trend over time in the prevalence of overweight was seen in 12-year-old girls with the least educated mothers, whereas among the other girls no statistically significant change was seen over time.
Lioret *et al. *2009 [43]	France	1998–2007	Comparison of 2 cross sectional food consumption surveys (INCA 1, 1998/9 and INCA 2, 2006/7)	N = 1034 3–14 year olds	Health of household occupation and level of education; variables describing household wealth.	Though the prevalence of childhood overweight was significantly and inversely correlated to all SES indicators, there was no change in the strong inverse SES gradient of childhood overweight over time, from 1998 to 2007
Romon *et al. *2005 [45]	France	1989–1999	Comparison of cross-sectional surveys of children in the final year of nursery school in the city of Lille, 1989 and 1999	N = 705 in 1989 and 1258 in 1999) 5 year old nursery children	Occupation of the father or occupation of the single parent. Occupation divided into four classes: (I) Professional and managerial occupations; (II) Intermediate occupations (Employees, own account workers); (III) lower occupations (technical, semi routine and routine); (IV) unemployed	The change in the prevalence of overweight over time differed as a function of social class: in 1989 there were no significant differences between social classes whereas in 1999 there was a clear difference between classes both for overweight (*p *= 0.0005) and obesity (*p* = 0.04).
Semmler *et al. *2009 [47]	UK	1998–2005	Subsample from the Twins Early Development Study. Comparison of socio-demographically and geographically matched families on the basis of parental weight status	N = 346 children aged 4 (1998/9) and followed up 7 years later	‘Family SES’ refers to maternal education: Lower SES = GCSE or lessHigher SES = A level or above	The change in adiposity from ages 4 to 11 differed as a function of familial SES: at age 11, significantly more children from lower SES families (29%) than higher families (17%) were overweight (*P* = 0.046).
De Spiegelaere *et al. *1998 [49]	Belgium	1988–1994	Retrospective cohort study on a sample of 12–15 year old adolescents born between 1976 and 1979, taken from six school medical centres in Brussels region.	N = 2607 adolescents aged about 12 at 1st examination and about 15 years at the 2nd, from five social groups	Five social groups were defined according to parents’ professions (Upper management/professionals, white collar workers, active self-employed and technicians, active manual workers, unemployed and dependent on state assistance) and the status of their activity (active or inactive: out of work, invalid, *etc.*)	Social inequalities in obesity increased between the first and second examination approximately two years apart: for the whole sample, there was a significant increase in the prevalence of obesity in the least-favoured social group (+3.3% (+/− 2.7%)).
Stamatakis *et al. *2010 [48]	UK	1997–2007	Ten cross-sectional surveys, the Health Survey for England (annual, omitting 1999)	5–10 year old children	Socioeconomic position was a composite score based on family household income (quintiles) and social class scales (I, II, III manual, III non manual, IV and V), based on occupation of the head of the household.	The gradient in the prevalence between high and low income groups was significant for overweight in boys (*p* = 0.04) and girls (*P* = 0.003) in 2006/7 and for obesity in girls in 2002/3 (p = 0.001), 2004/5 (*p* = 0.005) and 2006/7 (*p* = 0.04). The socioeconomic position (SEP) score gradient also increased over time, reaching significance in 2006/7 for boys’ overweight (*p* < 0.001) and obesity (*p *= 0.002) when obesity prevalence in the low SEP score group was twice than that in the higher groups.

In young children, psychosocial and socioeconomic deprivation are independently important risk factors for obesity [72], with a higher likelihood of reported poor psychological well-being and lower life satisfaction, which are in turn associated with risk of weight gain [56,73]. 

The evidence on the role of, and inequalities in, food and nutritional status is well-established. Household food insecurity has been linked to childhood and adolescent overweight [74,75], and has a dietary pattern: children living in more deprived circumstances tend to eat less fresh fruit and vegetables but more sugar and sweets, fats, processed meats, salty snacks and soft drinks compared with those from higher income households [73,76]. Cribb *et al. *[77] found that lower maternal education was associated with unhealthier food choices in 10 year old British children. Recent data from the HBSC study [35] and the DAFNE study (24 European countries) [78] confirm that lower socioeconomic status is associated with more frequent and higher availability of soft drinks in the household, and higher reported consumption levels. There may also be a social gradient in sedentary behaviours, physical activity and access to physical activity facilities in young people [35,79,80,81,82,83,84]. A clear social gradient in sedentary behaviour is seen in recent data from France where children of workers will spend twice as long watching television and playing video games than children of fathers with a management level job [85]. 

Families confronting poverty and insecurity will face competing priorities and constraints that may lead to unintended disinvestment in health and healthy behaviours [50]. Thus the widening social gradient in childhood obesity in some populations may partly be due to healthy eating and physical activity being considered a low priority in deprived households [48,86,87,88]. Moreover, in many settings, the cost of healthy eating may be considerably higher than the alternatives, especially where access to fresh supplies is difficult, as is the case in many deprived areas [89]. 

### 4.3. Implications for Further Research

Studies are needed to identify groups that are particularly vulnerable to obesity across Europe, to provide a better understanding of the factors and circumstances promoting this inequality [90], and whether and how they are changing over time. Research on the socioeconomic gradient in objectively measured health behaviours in children (as risk factors for obesity) is warranted. Further analyses of repeated surveys will continue to clarify the relation between socioeconomic status and obesity and how it changes over time. Longitudinal studies would provide further information, from a life-course perspective, on the health consequences of excess body weight, and on whether these differ between countries. Both quantitative and qualitative research are needed to understand the complexities of the association between socioeconomic status and obesity. Moreover further research taking into consideration the multiple socioeconomic indicators of overweight as well as particular risk factors will be essential if we are to understand fully the relationship between social status and child obesity [7,48,91]. Socioeconomic status can be measured by many indicators [26]. Child socioeconomic status is much harder to ascertain than adult status as it is usually reported either by the parent or the child, and can include parental education or occupation, family income or a composite measure derived from these indicators (such as a deprivation score [46]. In a review by Shrewsbury & Wardle [34], parental education showed the most consistent inverse relationship with the risk of child obesity, as compared to parental occupation or income. The authors drew on a theoretical framework proposed by Sobal wherein socioeconomic indicators operate differently to influence weight gain. Thus, where parental education is relatively stable and may influence knowledge and beliefs of children, parental occupation or income status may be less stable and may influence lifestyle and access to resources [92]. Complicating the relationship further are factors such as ethnicity. As in adults, there appears to be evidence that certain minority ethnic child populations are disproportionately vulnerable to obesity: a 2005 German study of migrant children entering primary school found substantial differences in overweight prevalence according to immigrant status. The researchers found that mothers’ educational status explained much of the variation, and that a behavioural factor—a high level of use of television during the week—in combination with the level of maternal education accounted for virtually all the ethnic differences [93]. 

## 5. Conclusion

There is good evidence for an association between childhood obesity and deprivation in Europe. The reasons underpinning this association, and the apparent widening social gradient in overweight in some studies, are not straightforward. However, it is likely that they include factors acting throughout the life course, as well as differential exposure to risk factors and, potentially, differential effects of exposure to such factors, both shaped by the social circumstances in which children find themselves. However, on the basis of what is already known about child development, it is likely that effective strategies will include investment in promoting health in early childhood (including *in utero*) as a means to enhance cognitive development and learning potential, embedded within comprehensive policies that consider all aspects of the environments within which children grow and develop. 
